# Structure–Property Studies on a New Family of Halogen Free Flame Retardants Based on Sulfenamide and Related Structures

**DOI:** 10.3390/polym8100360

**Published:** 2016-10-14

**Authors:** Teija Tirri, Melanie Aubert, Weronika Pawelec, Anton Holappa, Carl-Eric Wilén

**Affiliations:** Faculty of Science and Engineering, Chemical Engineering, Polymer Technology and Center for Functional Materials, Åbo Akademi University, Biskopsgatan 8, Abo 20500, Finland; Teija.tirri@abo.fi (T.T.); maubert@abo.fi (M.A.); WPawelec@uclan.ac.uk (W.P.); anton.holappa@gmail.com (A.H.)

**Keywords:** sulfenamide, radical generator, halogen free, flame retardant, polypropylene, polystyrene

## Abstract

A wide variety of molecules containing S–N or S–N–S cores were synthesized, and their flame retardant properties in polypropylene (PP), low density polyethylene (LDPE) and polystyrene (PS) were investigated. In addition, polymers or oligomers bearing the sulfenamide functionality (SN) were also synthesized. It was shown that this radical generator family based on sulfenamides is very versatile in terms of structural modifications, and the thermal decomposition range can be easily adjusted by changing the *R* groups attached to the core. The thermal stabilities of the different sulfenamides were examined by thermogravimetric analysis (TGA). Radicals generated by the homolytic cleavage of the S–N or S–N–S bonds at an elevated temperature can effectively interact with the intermediate products of polymer thermolysis and provide excellent flame retardant properties. The choice of most suitable SN-structure varies depending on the polymer type. For polypropylene DIN 4102-1 B2 and UL94 VTM-2 classifications were achieved with only 0.5 to 1 wt % of sulfenamide, and, in some cases, no flaming dripping was observed. Also for LDPE thin films, sulfenamides offered the DIN 4102-1 B2 rating at low dosage. In the case of polystyrene, the very stringent UL94 V-0 classification was even achieved at a loading of 5 wt % of sulfenamide.

## 1. Introduction

In response to the urgent need to develop new high-performance and halogen-free fire resistant materials with low environmental impact [1], different alternative flame retardant (FR) systems have been introduced. In many applications, traditional inorganic, non-halogenated flame retardants such as ammonium polyphosphate (APP) and aluminum or magnesium hydroxides (ATH, MDH) are widely used, but there is still a need to find more effective solutions. A vast majority of the proposed solutions are based on different reactive or additive organophosphorus or nitrogen containing additives that can act in gas phase and/or in condensed phase [2,3,4,5]. In addition, various families of radical generators have been prepared and their viability in a series of polymer applications has been recently demonstrated [6,7,8,9,10,11,12,13].

Most plastic materials require specific FRs for optimal fire safety; furthermore, FR materials must often fulfill very application-specific fire safety demands in order to pass various fire safety standards established for plastic components. Coating, construction, textiles, electric/electronic (E&E) and transportation industries have additionally various requirements on the additives used in their polymer materials; temperature stability, being solid or liquid, viscosity, polarity and miscibility are only a few properties which need to be matched between a suitable FR, the polymer, the production process and the product. Hence, the optimal flame retardant solution and the obtained level of flame retardancy depend on the polymer type and application. In this respect, flame retardants that can be compounded at temperatures ≥230 °C, have high hydrolytic stability, and do not require high loadings (below 10% by weight) in order to achieve compliance with UL94 V-0 would be highly desired. In addition, they should have a reasonable price compared to the halogen-containing flame retardant systems.

Along with phosphorus, nitrogen and silicon, sulfur is one of the known flame retardant elements, but compared to the others, sulfur containing compounds have been much less extensively studied as flame retardants. However, disulfide based radical generators alone or in combination with phosphorus compounds have shown good flame retardant effects both in polypropylene (PP) and polystyrene (PS) [12,14]. Replacing P=O bonds with P=S in 9,10-dihydro-oxa-10-phosphaphenanthrene-10-oxide (DOPO) enhanced gas phase FR activity in epoxy matrix [15]. An approach to combine P, N and S to an effective char forming FR for thermoplastic polyurethane has also been reported recently [16].

We have discovered that sulfenamides, a specific group of compounds containing a bond of divalent sulfur and trivalent nitrogen, which by homolytical cleavage generate aminyl and thiyl radicals, provide excellent flame retarding properties to polymer substrates [17]. Sulfenamides (SN) contain two reactive centers, and can be attacked both by nucleophiles on S and by electrophiles on N. Formation of sulfenamides was reported already in 1911 by Zincke [18]. Since then, sulfenamides and related structures have been extensively studied as intermediates in organic and peptide synthesis and used in various applications including pharmaceuticals, fungicides and rubber vulcanization [19,20,21]. One of the very attractive features of sulfenamide compounds is their versatility; by changing the *R* groups attached to S and N, very different kinds of chemical and physical properties can be obtained. Hydrophobicity, thermal properties and stability in different chemical environments can be tailored to fit the application-specific requirements. Both small molecules and polymers can be easily synthesized. The synthesis of first polysulfenamides using secondary diamines and dithiosuccinimid sulfenyl transfer agent was described in 2011 [22,23,24]. These polymers are based on aliphatic and cycloaliphatic constituents, and they readily degrade in water. In vivo and in vitro toxicological studies of the fabricated polysulfenamide microparticles showed little or no adverse effects, and were therefore proposed to be a new class of biodegradable polymers for drug delivery.

Eichhorn has shown already in the 1960s that certain radical generators such as peroxides, benzothiazole, sulfenamides, disulfides and bibenzyl compounds can be used as synergists with brominated flame retardants in vinyl aromatic polymers [25,26]. Eichhorn reported that radical precursors including sulfenamides trigger the decomposition of halogenated FRs, whereby halogen radicals are rapidly liberated which effectively interrupts the combustion process. Thus, according to Eichhorn, without the addition of halogenated flame retardant, self-extinguishing of the flame will not occur if only a free radical generator per se is employed. To the best of our knowledge, the use of sulfenamides and related structures as independent fire protection agents is not found in prior literature.

**Figure 1:**
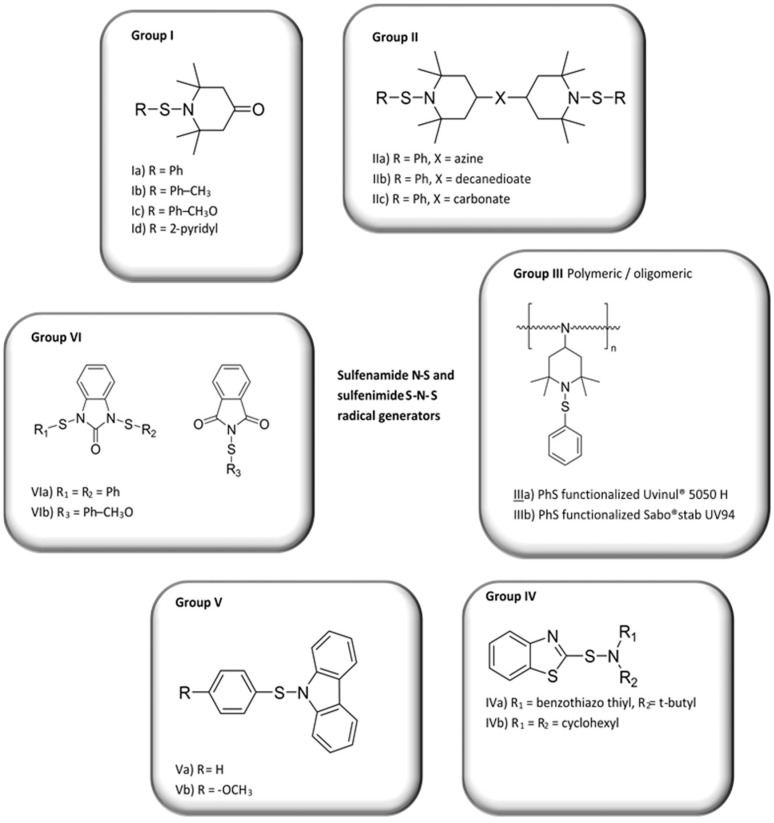
Grouping of sulfenamides.

The excellent flame retarding action of various sulfenamide compounds on polypropylene, low density polyethylene and polystyrene when exposed to small heat source is reported herein. The novel sulfenamide flame retardants have been divided into six groups based on their structure, as shown in Figure 1.

Even polymeric structures with sulfenamides attached as pendant groups are presented. In general, oligomeric or polymeric plastic additives are sometimes preferred, especially in pipe and textile applications, since polymeric additives are less prone to physical loss compared to low molecular weight ditto, when in contact with aggressive service environments such as organic solvents and/or detergents.

## 2. Experimental Section

Synthesis and characterization of the SN compounds Ia—VIb are described in a recently filed patent [17]. All chemicals used were of reagent grade and purchased from Aldrich (Helsinki, Finland), ABCR (KArlsruhe, Germany) or Wako Chemicals GmbH (Neuss, Germany), except for the commercial samples obtained from BTC Europe (Uvinul^®^ 5050 H, Irganox^®^ B 225, Rhein, Germany), Songwon Industrial Co. (Sabo^®^stab UV 94, Ulsan, Korea) and Flexsys (Santocure TBSI, now part of Eastman, Capelle aan den IJssel, Holland). The Santocure TBSI (Compound IVa) was purified by trituration with isopropanol before use. Irganox^®^ B 225 is a processing and long-term thermal stabilizer, a blend of 50% tris(2,4-ditert-butylphenyl) phosphite and 50% pentaerythritol tetrakis[3-[3,5-di-*tert*-butyl-4-hydroxyphenyl]propionate].

^1^H and ^13^C NMR spectra were recorded on a Bruker Avance 600 (^1^H 600.1 MHz, ^13^C 150.9 MHz) (Billerica, MA, USA). Thermogravimetric analyses (TGA) were performed using an SDT Q600 apparatus from TA Instruments (New Castle, DE, USA) under N_2_ atmosphere at a heating rate of 10 °C/min.

Flame retardants were melt mixed in polypropylene (PP, MOPLEN HP500N from LyondellBasell, Rotterdam, Holland, melt flow rate (MFR) 12 g/10 min (230 °C/2.16 kg)) and low density polyethylene (LDPE, Borealis Polymers CA7230, Kulloo, Finland, MFR 4.5 g/10 min (190 °C/2.16 kg)) in a Haake Rheocord melt blender (Waltham, MA, USA) (60 rpm). Polypropylene was melted first for 1 min at 210 °C, where after processing aids Ca-stearate (Baerlocher, Unterschleißheim, Germany, 0.05 wt %) and Irganox^®^ B 225 (0.3 wt %) were added. After one minute, flame retardant was added and the mixing was continued for five minutes. LDPE was processed similarly at 190 °C but without processing aids. Test films (200 μm and 1mm) were compression molded in a hot press at 210 °C for PP and at 135 °C for PE. Polystyrene (GPPS 25SPI, LG Chemicals,Frankfurt, Germany) formulation was compounded by twin screw extrusion with die temperature set at 200 °C, followed by injection molding at 200 °C into 1.6 mm thick strips (127 × 12.7 mm^2^) for UL 94 tests. All samples were conditioned three days prior to testing (23 °C, 50% RH).

The flame retardant effect was investigated in accordance with a modified DIN 4102-1 B2. The flame application time was 15 s with a 40 mm flame height and edge ignition. Polystyrene samples were tested according to UL94 standard [27].

## 3. Results and Discussion

In the course of this study, a series of radical precursors based on sulfenamide derivatives were prepared in order to explore their utility as novel flame retardants for polyolefins. The prepared sulfenamide compounds were divided into six groups based on their chemical structures and their thermal stabilities were examined by TGA studies.

### 3.1. Synthesis of Sulfenamides

**Figure 2:**
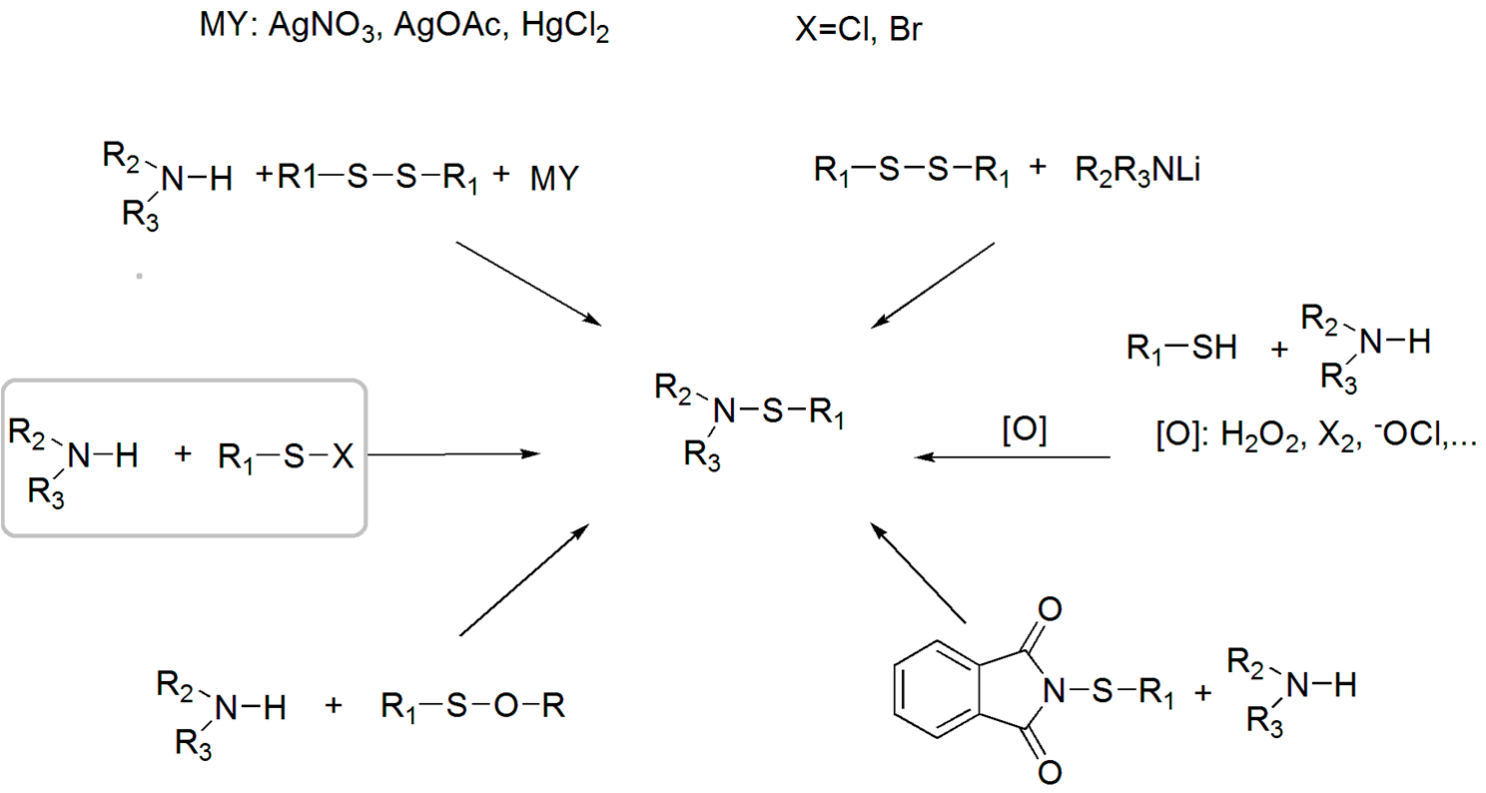
General synthetic methods developed for generating sulfenamide derivatives.

The general synthetic pathways for the preparation of sulfenamides have been reviewed in the literature [19,28,29,30,31,32,33]. The most common synthetic approaches to sulfenamides are depicted in Figure 2.

**Scheme 1:**
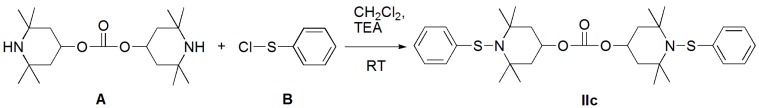
Synthesis of bis(2,2,6,6-tetramethyl-1-(phenylthio)piperidin-4-yl) carbonate.

The reaction of alkylthionyl chloride with an amine as highlighted in Figure 2 was used as a facile synthetic route for the generation of the targeted sulfenamide derivatives. Either excess of the same amine or triethyl amine was used to scavenge hydrogen chloride that was released during the reaction. The alkylsulfenyl chlorides were prepared from the corresponding disulfides or thiols by chlorination with Cl_2_ or SO_2_Cl_2_ in dry aprotic solvents. As an example, synthesis of the compound IIc is described here (Scheme 1).

#### 3.1.1. Bis(2,2,6,6-tetramethylpiperidin-4-yl) Carbonate (Diamine A)

The diamine A was prepared by transesterification reaction between diphenyl carbonate and 2,2,6,6-tetramethylpiperidinol. Under argon atmosphere, 2,2,6,6-tetramethyl-4-piperidinol (10 g, 64 mmol), diphenyl carbonate (6.8 g, 32 mmol) and potassium carbonate (0.58 g, 4 mmol) were dispersed in mineral spirits (65 mL). The reaction mixture was refluxed at 180 °C for 8 h. The mixture was left to cool down to 50 °C and washed with warm water (3 × 60 mL). White precipitate appeared upon cooling down to RT, which was filtrated off and dried under reduced pressure at 90 °C to remove residues of water and phenol. Pure product was obtained in 51% yield, 5.5 g.

^1^H NMR (CDCl_3_): 5.04 (m, CH, 2H), 2.04 (d, CH_2_, 2H), 2.02 (d, CH_2_, 2H), 1.25 (s, CH_3_, 12H), 1.20 (m, 4H), 1.17 (s, CH_3_, 12H).

^13^C NMR (CDCl_3_): 154.28, 72.70, 51.76, 44.05, 35.01, 28.81.

#### 3.1.2. Benzenesulfenyl Chloride (B)

A three-necked reaction flask equipped with a reflux condenser fitted with an acid gas receiver (15% NaOH solution) was charged with phenyl disulfide (10 g, 46 mmol) and dry CH_2_Cl_2_ (100 mL). The solution was cooled down to −18 °C and Cl_2_ gas was passed in over 20 min. The solution got a strong orange-red color. The cooling bath was allowed to warm up to −5 °C, and then the flask was removed from the bath and the solution was stirred gently for 30 min under argon flow. The complete conversion of the disulfide into phenylsulfenyl chloride was confirmed by ^1^H NMR without solvent removal.

^1^H NMR (10% CDCl_3_): δ 7.39–7.45 (m, 3H), 7.65–7.7 (m, 2H).

#### 3.1.3. Bis(2,2,6,6-tetramethyl-1-(phenylthio)piperidin-4-yl) Carbonate (Structure IIc)

Diamine A (0.5 g, 1.5 mmol) was dissolved in dry dichloromethane (5 mL) in an argon atmosphere, and triethylamine (0.52 mL, 3.6 mmol) was added. Then, benzenesulfenyl chloride in CH_2_Cl_2_ (4.07 mL, 3.7 mmol) was added dropwise. After complete addition, the reaction mixture was stirred at room temperature for 2 h. The organic phase was washed with water (3 × 10 mL), dried over Na_2_SO_4_ and evaporated. The product was recrystallized twice from diethyl ether and the title compound was obtained in 84% yield (0.7 g).

^1^H NMR (CDCl_3_): 7.24 (m, CH, 8H), 7.06 (m, CH, 2H), 5.04 (m, CH, 2H), 2.19, 2.16 (d, CH_2_, 4H), 1.80 (m, CH_2_, 4H), 1.36 (s, CH_3_, 12H), 1.28 (s, CH_3_, 12H).

^13^C NMR (CDCl_3_): 154.2, 145.0, 128.0, 124.1, 121.7, 70.7, 61.2, 45.3, 32.2, 25.5.

### 3.2. Thermal Degradation Behaviour and Tuning of Sulfenamide Structure

**Scheme 2:**
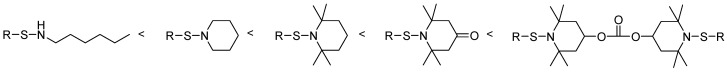
Substituent effect on thermal stability of sulfenamide compounds.

Thermal stability of sulfenamides is affected by the *R*-groups attached to S (R1) and N (R2, R3). Low basicity and increased number of substitutions of the nitrogen atom increase the stability. Thermal stability decreases, if R1 contains groups capable of anchimeric assistance in cleavage of the S–N bond, whereas electron withdrawing substituents on R increase the thermal stability [33]. Delocalization of the nitrogen non-bonding electron pair decreases the basicity of amine, and therefore electron withdrawing groups on R2 and R3 decrease the likelihood of N protonation. Effective delocalization can lead to inversion of the trigonal pyramidal geometry of N towards planar trigonal. The latter geometry is beneficial also for the stabilization of the nitrogen radical formed during the cleavage of the S–N bond. Heterocyclic aromatics containing nitrogen atoms bonded to carbonyl groups, as in phthalimide, tend to be highly thermally stable. Nitrogen atoms in aromatic heterocyclic structures, as in group V compounds, or bonded to carbonyl groups, as in group VI compounds, tend to have planar geometry. In addition, sterical hindrance can protect the nitrogen. The effect of structure on thermal stability is illustrated in Scheme 2 for a series of SN-compounds.

Short S–N bond length correlates to the good thermal stability. If the distance between sulfur and nitrogen atoms is less than normal 1.73 Å for a single bond, the bond exhibits some π-character [34,35,36]. Thermal decomposition behaviour of sulfenamide accelerators has been extensively studied for rubber vulcanization. To achieve high vulcanization efficiency while simultaneously maintaining long vulcanization delay (scorch time), sulfenamides with calculational S–N bond length less than 1.67 Å and high stability of the dissociated aminyl radicals are particularly suitable [37]. Consequently, the thermal stability is high, but once the SN-bond decomposes, it does not easily recombine.

**Figure 3:**
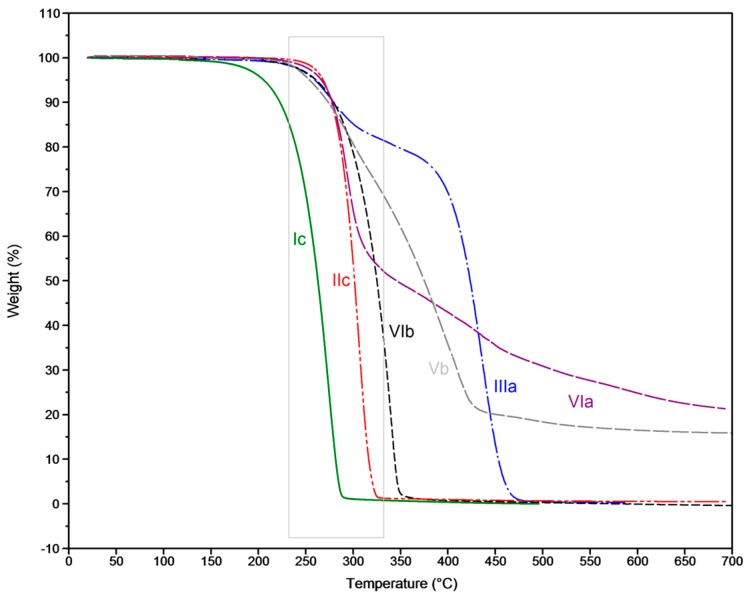
Collected thermogravimetric analysis (TGA) graphs of sulfenamides denoted Ic, IIc, IIIa, Vb, VIa and VIb. The optimal temperature range for flame retardant (FR) decomposition between polypropylene (PP) processing (230 °C) and ignition temperatures (250–360 °C) is marked with a grey border.

Thermal weight loss curves for sulfenamides Ic, IIc, IIIa, Vb, VIa and VIb are shown in Figure 3. Suitable decomposition temperature range for flame retardants used in PP formulations has been marked with a grey border line. The sulfenamide should not decompose during PP processing at temperatures up to 230 °C. However, it should be activated in the temperature range of 250–360 °C when the pyrolysis products of PP (aliphatic saturated and unsaturated hydrocarbons) from the surface are becoming sufficiently vaporized for ignition to take place [38]. In this context, it can be stated that all of the tested sulfenamide compounds exhibited appropriate thermal properties for potentially being suitable flame retardants for PP. Unlike the other sulfenamides, compounds Vb and VIa had relatively high char residues of 15–20 wt % at 700 °C, which was related to their structural capability to form polyaromatic condensation products.

### 3.3. Flame Retardancy Tests

All polypropylene, low density polyethylene and polystyrene formulations containing various sulfenamides as flame retardant additives successfully pass the DIN 4102-1 B2 classification. According to the DIN 4102-1 standard, a 20 mm flame is applied for 15 s, and the average burning time when the flame is removed is recorded. However, in many cases, the sample self-extinguished already before the flame was removed, and only melting was detected after that. Therefore, in Table 1, Table 2 and Table 3, burning time is given from the application of flame, not from the flame removal. In addition, a 40 mm flame was used in order to better differentiate between the flame retardant efficacies of different sulfenamide derivatives. DIN 4102-1 B2 classification is comparable with class E in the EN 13501-1 “Fire classification of construction products and building elements”.

The first group of synthesized sulfenamides based on 2,2,6,6-tetramethylpiperidinone, i.e., Ic–Id, with different thioaryl groups exhibited all very similar and short burning times in thin polypropylene films of 200 μm (Table 1).

Additional fire tests were performed using Ic in thick polypropylene plaques of 1 mm in accordance with DIN 4102-1 standard and films of 300 μm based on a UL94 VTM test standard. The results revealed that even polypropylene plaques and films containing 0.5 wt % of Ic passed the criteria’s set forth for DIN 4102-1 B2 (see Table 2) and UL94 VTM-2 classifications, respectively.

The prepared LDPE samples flame retarded with Ia and IIa passed the DIN 4102-1 B2 test. Both burning time and damage length were, however, somewhat longer than for the corresponding PP thin samples (Table 3).

The second group of sulfenamides was based on bis(2,2,6,6-tetramethyl piperidinyl) that are less volatile than group I sulfenamides. The samples self-extinguished even before flame removal (Table 1). The recorded damage lengths above 55 mm were mainly caused by polymer melting. The thermal stabilities were dependent on the linker moiety X and increased in the following order: IIb < IIa < IIc. The compound IIc was chosen as the most promising candidate in this series due to its excellent flame retardant efficacy, high thermal stability, excellent processing behavior and clarity of the produced polypropylene film.

**Figure 4:**
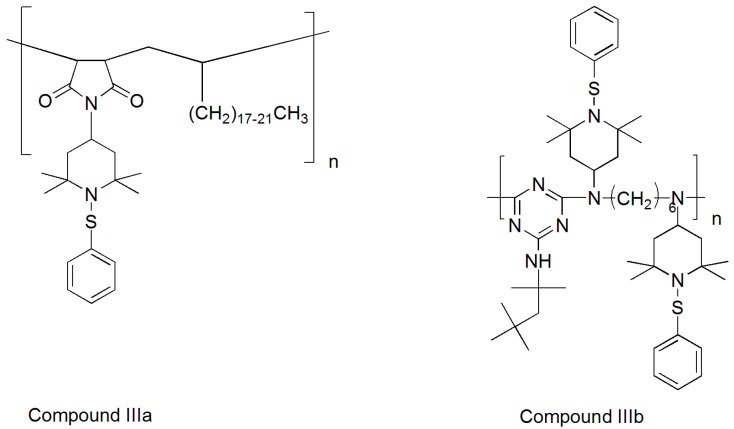
Tentative structures for polymeric compounds IIIa and IIIb.

The third group of sulfenamides consisted of polymeric sulfenamide structures. Tentative structures for the reaction products of Uvinul^®^ 5050 H and Sabo^®^stab UV 94 are shown in Figure 4. Similar thermal stabilities and anti-flame properties were recorded for both polymeric compounds IIIa and IIIb (Table 1). The result demonstrates that polymeric sulfenamides are also able to effectively impart flame retardancy to polypropylene films.

Sulfenamides belonging to group IV contain benzothiazole moieties, and they were commercially available. Traditionally, these types of sulfenamides are used as non-nitrosamine generating accelerators in rubber vulcanization. Compound IVb was thermally more stable than most sulfenamides used in the rubber industry, and was therefore selected to be the more potent candidate as a flame retardant for PP within this family of sulfenamides. Interestingly, this structure passed the DIN 4102 B2 flammability test, but it was not as effective as the 2,2,6,6-tetramethylpiperidine based sulfenamides of group I (Table 1).

One of the most impressive results was obtained when 5 wt % of sulfenimide IVa was used in 1.6 mm thick polystyrene sticks (Table 4). Thus, the stringent UL94 V-0 rating was successfully reached. The reference virgin PS burned very vigorously with a high flame. In contrast, polystyrene containing IVa exhibited excellent flame retardant properties with a total burning time of only 4 s for five sticks.

**Figure 5:**
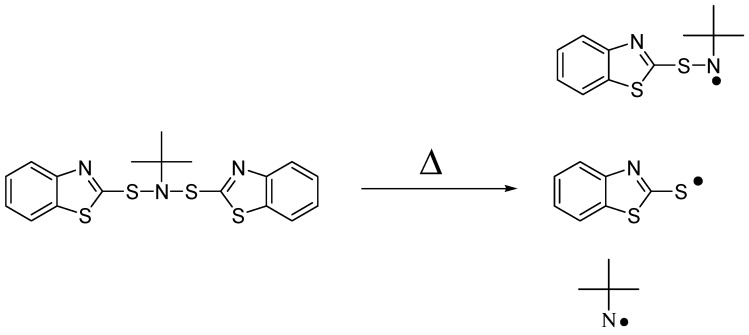
Possible radicals generated by thermal decomposition of IVa.

The fact that IVa is such an effective FR in polystyrene was attributed to generation of different radicals depicted in Figure 5, which by a cascade of different reactions take part in degradation enhancement of polystyrene as earlier described in the literature [14,39,40]. When thiyl and amine radicals are generated by thermal cleavage, they can abstract hydrogen from polystyrene backbone, form intermediate reaction products where sulfur species (e.g., polysulfides) are attached to polystyrene and take part in various kind of chain transfer and telomerization reactions. Sulfur catalyzed recombination of flame radicals can also occur.

In order to study amine moieties with a very high charge dissipation capacity, carbazole based sulfenamide derivatives of group V were synthesized. The thermal stability of compounds Va and Vb was expected to be high due to planarity of N. Both compounds exhibited excellent flame retardant performance at a loading of 1 wt % without any flame dripping. Compound Vb had slightly higher thermal stability and showed better flame retardancy than compound Va. Both compounds also passed the criteria for UL94 VTM-2.

The sixth group of sulfenamides contained imide and imidazole moieties. In the imide and imidazole derivatives, the N atom is next to electron withdrawing carbonyl groups. In theory, this type of sulfenamides should have high thermal stabilities and excellent free-radical quenching capabilities. It was found that both VIa and VIb could effectively improve the flame retardancy of polypropylene films.

## 4. Conclusions

We have shown that, when exposed to a small ignition source, various sulfenamide derivatives can help to self-extinquish films of PP, LDPE and PS already at a very low dosage ranging from 0.5–5 wt %. In general, at these very low loadings, various radical generators do not provide sufficient fire protection when the polymers are exposed to more harsh fire scenarios such as in cone calorimeter tests alone, but they can be used to enhance protection given by intumescent or char forming systems, as will be shown in our subsequent paper. However, fire often starts by a small ignition source. If the material around it self-extinguishes, it can effectively prevent fire from spreading. Studies related to their flame retardant mechanism are also under way.

## Figures and Tables

**Table 1:** Temperature at 5% mass loss (T_5%_) for pure flame retardant (FR) and flame retardancy results for polypropylene (PP) with a given amount of added FR.

Flame retardant	TGA weight loss T_5%_	DIN 4102-1 B2 ^1^			
		FR amount (wt %)	Average damage length (mm)	Average burning time (s) ^2^	(Pass/Fail)
Reference PP		0	190	39	Fail
Ia	ND ^3^	0.5	71	15	Pass
Ib	ND	0.5	56	8	Pass
Ic	ND	0.5	68	9	Pass
Id	ND	1.0	64	16	Pass
IIa	264	1.0	102	12	Pass
IIb	248	1.0	81	10	Pass
IIc	269	1.0	76	11	Pass
IIIa	254	1.0	80	12	Pass
IIIb	259	1.0	83	18	Pass
IVb	234	0.5	98	23	Pass
Va	238	1.0	75	12	Pass
Vb	254	1.0	61	7	Pass
VIa	266	0.5	67	14	Pass
VIb	265	1.0	69	18	Pass

^1^ DIN 4102-1 B2 classification is comparable with class E in the EN 13501-1 “Fire classification of construction products and building elements”. ^2^ Burning time is calculated from the application of flame. Flame application time 15 s, edge ignition, flame height 40 mm. PP film thickness 200 μm. ^3^ ND = not determined. TGA = thermogravimetric analysis.

**Table 2:** Flammability results for 1 mm thick PP plaques with a given amount of added FR.

Flame retardant	DIN 4102-1 B2			
	FR amount (wt %)	Average damage length (mm)	Average burning time (s) ^1^	Pass/Fail
Ic	1.0	43	20	Pass
IIb	1.0	43	21	Pass
IIc	1.0	52	30	Pass

^1^ Burning time is calculated from the application of flame. Flame application time 15 s, edge ignition, flame height 40 mm.

**Table 3:** Flammability results for low density polyethylene (LDPE) with a given amount of added FR.

Flame retardant	DIN 4102-1 B2			
	FR amount (wt %)	Average damage length (mm)	Average burning time (s) ^1^	(Pass/Fail)
Reference LDPE	0	190	37	Fail
Ia	0.5	108	30	Pass
IIa	0.5	133	36	Pass
	1.0	122	28	Pass

^1^ Burning time is calculated from the application of flame. Flame application time 15 s, edge ignition, flame height 40 mm. LDPE film thickness 200 μm.

**Table 4:** UL 94 V test results for polystyrene.

Flame retardant	TGA weight loss T_5%_	UL 94 V		
		FR amount (wt %)	Total burning time (s) ^1^	Rating (V-0/Fail)
Reference PS	-	0	ND	Fail
Iva	194	5	4	V-0

^1^ Total burning time for five sticks of 1.6 mm thickness. UL94 V = Underwriters laboratories standard test for vertical specimens [27].
